# P-1675. BioFire Pneumonia Panel: An Evolving Asset in the Pursuit of Diagnostic Stewardship

**DOI:** 10.1093/ofid/ofaf695.1849

**Published:** 2026-01-11

**Authors:** Tripti Jain, Maryrose R Laguio-Vila, Ali S I Mohamed

**Affiliations:** Rochester General Hospital, Rochester, New York; Rochester Regional Health, Rochester, New York; Rochester General Hospital, Rochester, New York

## Abstract

**Background:**

The BioFire Pneumonia Panel (BF-PP) rapidly identifies respiratory pathogens and resistance genes within hours, compared to ≥48 hours for conventional cultures and even longer for antibiotic susceptibility testing. In the interim, patients are often empirically treated with broad-spectrum antibiotics or may be inadequately covered for resistant organisms.

**Figure 1:**
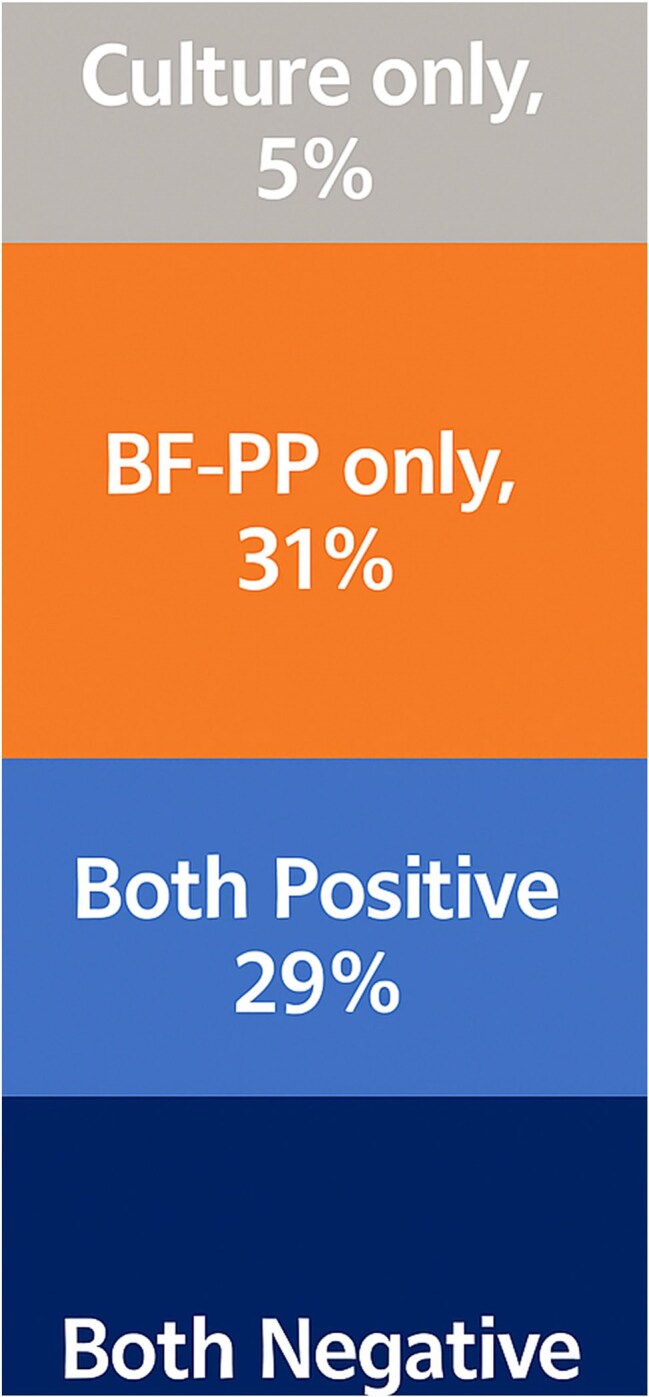
BF-PP vs. Sputum Culture Positivity Rate

**Figure 2:**
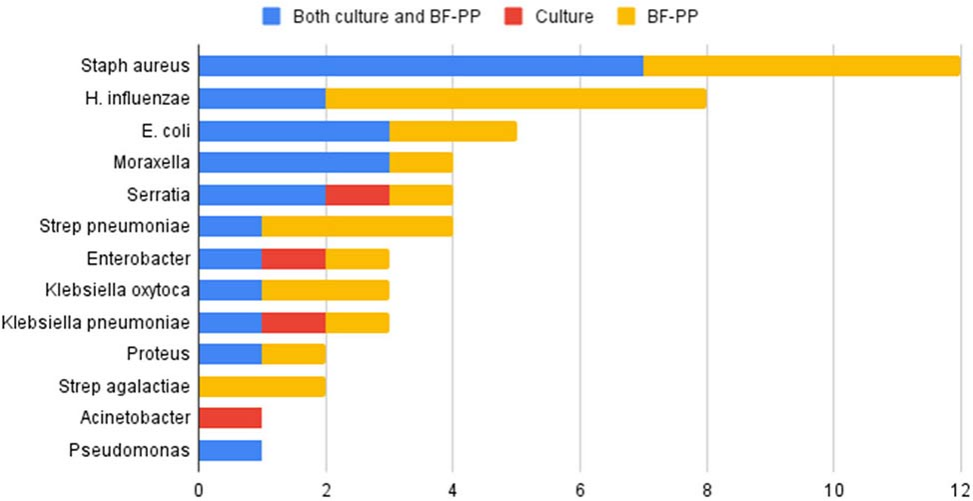
Organisms Isolated by Modality

**Methods:**

We conducted a retrospective chart review of all patients who underwent BF-PP testing at Rochester General Hospital between September 2023 and November 2024. BF-PP results were compared with standard respiratory cultures and classified as concordant positive/negative, BF-PP-exclusive positive, culture-exclusive positive, or undetectable by BF-PP. Post-result antibiotic changes were categorized as escalation, de-escalation, or no change.

**Figure 3:**
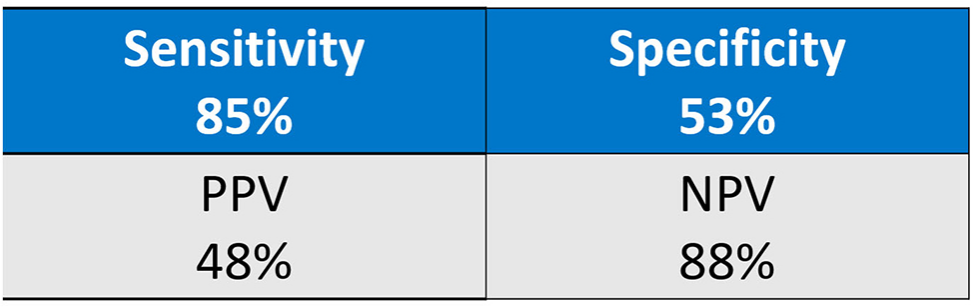
Test Performance Characteristics

**Figure 4:**
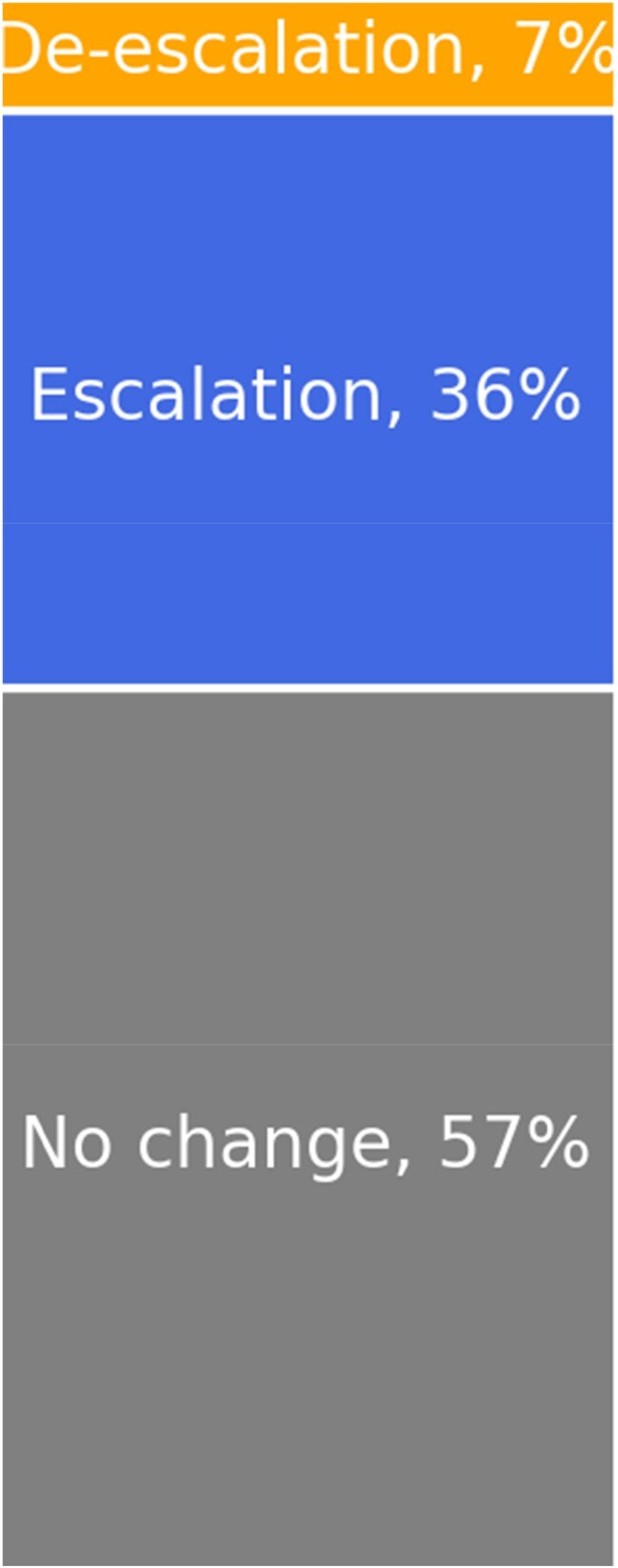
Antibiotic Change Following BF-PP Results

**Results:**

Of the 58 patients tested, one sample was excluded due to poor quality. Five patients (8.6%) had organisms not detectable by BF-PP (Candida spp. [n=3], Stenotrophomonas [n=2]). As many samples grew multiple bacterial organisms, the total number of pathogens identified (n=52) exceeded the number of positive samples (n=24). BF-PP and culture were concordantly positive in 23 cases (29%), while 28 samples (35%) were concordantly negative. BF-PP was exclusively positive in 25 samples (31%), and it missed culture-detected organisms in 4 samples (5%) (Figure 1). Figure 2 summarizes the organisms isolated by culture, BF-PP or both.

Sensitivity was 85%, and the NPV was 88% (figure 3). The BF-PP detected discordant methicillin resistance in one *S. aureus* case (reported as MSSA on culture), two concordant MRSA cases, and two concordant ESBL-producing *E. coli*. In four patients (7%), BF-PP-guided escalation ensured timely treatment with vancomycin or meropenem. Antibiotic de-escalation or discontinuation occurred in 36% of patients based on BF-PP results while more than half (57%) remained on the same antibiotics (figure 4).

**Conclusion:**

BF-PP offers rapid detection with moderately high sensitivity and moderate negative predictive value and supports earlier and more targeted antibiotic decisions in pneumonia management. However, conventional respiratory cultures remain essential for confirmation. Larger studies are warranted to validate these findings.

**Disclosures:**

All Authors: No reported disclosures

